# Assessment of prolonged safety and tolerability of erenumab in migraine patients in a long-term open-label study (APOLLON)

**DOI:** 10.1186/s10194-024-01860-w

**Published:** 2024-09-25

**Authors:** Hartmut Göbel, Eugen Schlegel, Kathrin Jaeger, Sonja Ortler, Lea Leist

**Affiliations:** 1 Schmerzklinik Kiel, Migräne und Kopfschmerzzentrum, Kiel, Deutschland; 2 Zentrum für Neurologisch-Psychiatrische Studien, ZNS GmbH, Siegen, Deutschland; 3 Novartis Pharma GmbH, Nürnberg, Deutschland

**Keywords:** Chronic migraine, Episodic migraine, Monoclonal antibody, Erenumab, Long-term safety, Tolerability, Drug holiday

## Abstract

**Background:**

Efficacy and safety of human monoclonal antibody erenumab used for migraine prophylaxis have been shown in clinical studies. APOLLON is an open-label, multi-center, single arm study, which permits dose adjustments of erenumab and includes an option for a drug holiday. The findings contribute to the accumulating long-term evidence regarding erenumab’s tolerability and safety profile in individuals experiencing episodic and chronic migraines.

**Methods:**

The study population consisted of adult patients with episodic or chronic migraine, who had successfully completed the HER-MES study (NCT03828539). Patients were treated with erenumab for 128 weeks at a flexible dose of either 70 mg or 140 mg. Treatment discontinuation attempts were allowed as voluntary single treatment interruption (‘drug holiday’) of up to 24 weeks.

**Results:**

701 patients were enrolled in APOLLON. The exposure associated incidence rate (EAIR) of adverse events (AEs) (*N* = 601) per 100 subject years was 101.71 (95% CI [92.28; 111.14]) meaning a patient could expect having about one adverse event per each year of treatment. EAIR was higher in females (*n* = 524, EAIR: 104.40, 95% CI [93.93; 114.86]) than in males (*n* = 77, EAIR: 86.55, 95% CI [65.39; 107.71]) and increased with initial monthly migraine days (MMD) and prior prophylactic treatment failures. A total of 155 patients discontinued erenumab treatment during open-label treatment phase. Of these, 29 were due to AEs (4.1% of total cohort) and out of these 65.5% (*N* = 19) were considered treatment-related. Safety parameters were in line with HER-MES data and did not reveal new safety signals. Drug holidays were realized by 108 patients (15.4%), of which 64.8% (*N* = 70) returned to treatment. The mean number of monthly headache days (MHDs), MMDs, and days with acute headache medication significantly increased during drug holiday. After resumption of erenumab treatment, a rapid reduction of the migraine parameters was observed.

**Conclusions:**

APOLLON provides long-term safety and tolerability data confirming the beneficial safety profile of erenumab over a period of 128 weeks. In addition, reversibility of migraine deterioration during drug holiday was shown and most patients returned to their treatment with similar response rates compared to initial treatment.

**Trial registration:**

ClinicalTrials.gov ID: NCT04084314 (https://clinicaltrials.gov/study/NCT04084314), First submitted: 2019-09-06.

## Background

Migraine is a neurological disorder that severely affects everyday life and is aggravated by comorbidities such as depression and cardiovascular diseases. The one-year prevalence of migraine in Germany is estimated to be 15% in women and 6% in men [1]. Patients now have access to a variety of therapies, and recently, specific preventive therapy targeting calcitonin gene-related peptide (CGRP) pathway has also become available. However, a real-world observational study enrolling patients with episodic (EM) and chronic migraine (CM) found, that the majority of participants, although suffering from an average of 9 migraine days per month, was naïve to preventive treatment, indicating the underuse of therapeutic options [2]. Another important finding of this study was that tolerability is a major factor to preventive treatment continuation and adherence. Many patients who used preventive therapy either discontinued within 6 months or changed dosing schemes and intervals on their own, mainly due to side effects [2].

With erenumab, an antibody targeting the CGRP receptor, that was introduced in 2018, an effective and well-tolerated preventive option is available [3–5] and could further improve preventive care for migraine patients. Recently the randomized controlled trial HER-MES compared the efficacy of erenumab to that of topiramate, a standard of care oral preventive drug. In this patient-centered setting, significantly more patients achieved a ≥ 50% reduction in monthly migraine days (MMD) from baseline with erenumab, which demonstrated a favorable tolerability and efficacy profile compared to topiramate [6]. Consequently, the updated guideline of the European Headache Federation recommends erenumab over topiramate as preventive treatment in individuals with EM or CM due to better tolerability. In addition, the guideline suggests that monoclonal antibodies targeting the CGRP pathway should be considered a first-line treatment option for migraine patients who require preventive treatment [7].

In the absence of a clear dose-dependent safety signal and a comparable overall efficacy trend between 70 mg and 140 mg erenumab [3], both dose groups are considered to offer a positive benefit-risk ratio for the chosen migraine patient population. As efficacy of 140 mg erenumab was proven in patients with ≥ 2 prior preventive migraine treatment failures [4, 8], certain patients might obtain an additional benefit from this dose [12]. Cumulating evidence supports the recommendation of 140 mg erenumab as starting dose for difficult-to-treat patients that benefit from the therapeutical gain with concurrently lacking a therapeutic penalty [9]. CGRP targeting in migraine represents a new and specific approach in migraine therapy. During the 5-year open-label treatment period of a Phase 2 EM prevention study, no increase in adverse events and no new safety signals over 5 years of exposure were observed [14]. But long-term data on safety and tolerability in a large group of patients is still accumulating. This also applies to pausing of treatment, which is recommended in the updated guideline of the European Headache Federation: in individuals with EM or CM a discontinuation attempt in the treatment with monoclonal antibodies targeting the CGRP pathway after 12–18 months of continuous treatment should be considered. However, in case of migraine worsening upon drug holiday, restarting of the treatment is suggested [7].

The objective of APOLLON was to evaluate long-term safety of erenumab in patients with EM and CM, allowing for both dose adjustment and an optional drug holiday.

## Methods

**Fig. 1:**
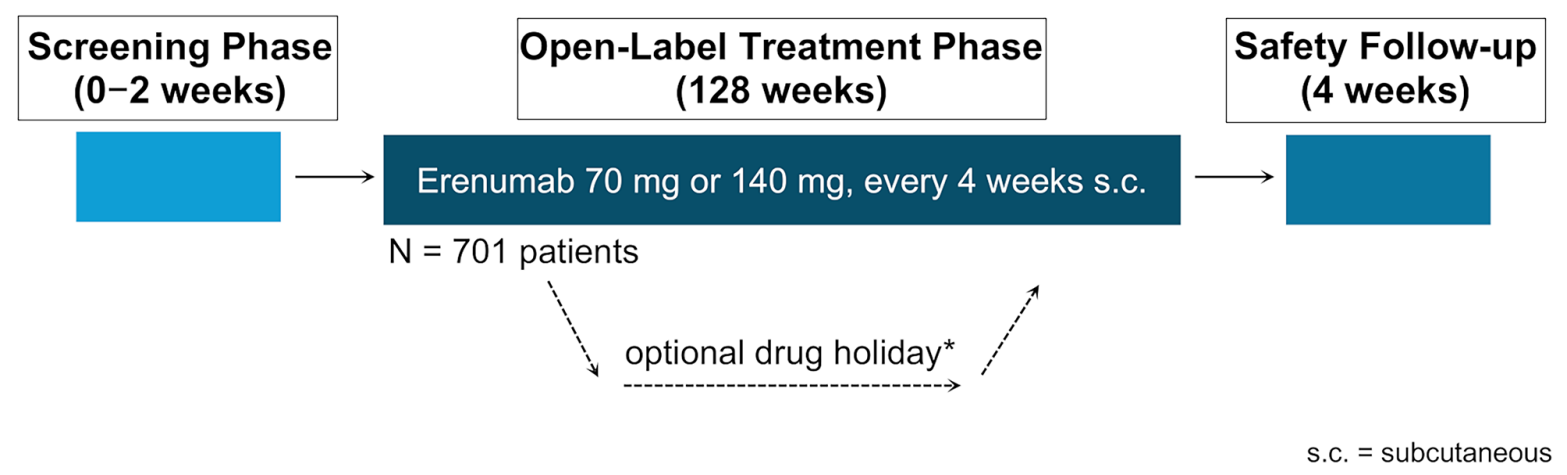
Study design. *Drug holiday of up to 24 weeks could be initiated after at least 12 weeks of treatment

APOLLON was an open-label, multi-center, single arm study conducted in Germany (79 study sites). Adult patients with a documented history of episodic (4 − 14 baseline migraine days) or chronic migraine (≥ 15 baseline headache days) who had been successfully randomized to and completed the clinical trial HER-MES (https://clinicaltrials.gov/ct2/show/NCT03828539) were eligible for participation. In order to implement the recommendation of the health technology assessment (HTA) body to include a full migraine population in the trial, a protocol amendment permitted patients with chronic migraine to be enrolled. At this time, the majority of patients of the total study population had already been enrolled, all of them patients with episodic migraine. Patients could be enrolled to APOLLON from two weeks until three months after their end of study visit in HER-MES. With entering the APOLLON study, participants and doctors were still blinded for previous treatment in HER-MES. Patients were eligible if they had not received prior prophylactic migraine treatment (naïve) or, due to lack of efficacy or tolerability, had failed or had not been suitable for up to three previous prophylactic treatments from the following: Metoprolol/propranolol, amitriptyline, and flunarizine. Furthermore, patients were excluded if they had a history of cluster headache or hemiplegic migraine, or were unable to differentiate migraine from other headaches. The use of any medication for migraine prophylaxis within five half-lives, or a device or a procedure within 1 month prior to the start of the baseline phase and during the study, was prohibited. APOLLON consisted of a 2-week screening period, followed by an open-label phase with erenumab treatment of 128 weeks and a follow-up safety monitoring (4 weeks) (Fig. 1).

Patients received erenumab subcutaneously at a dose of either 70 mg or 140 mg every 4 weeks. At study start, the patients were assigned to the latest administered dose in the HER-MES trial regardless of verum or placebo. During the open-label treatment phase, it was at the discretion of the treating physician to change the erenumab dose at each planned visit from 70 mg to 140 mg or vice versa. Voluntarily, a single treatment interruption (drug holiday) of up to 24 weeks could be initiated after at least 12 weeks of treatment (Fig. 1).

### Study endpoints

The primary endpoint was to evaluate the long-term safety of 70 mg and 140 mg erenumab in patients with EM or CM assessed by the exposure adjusted incidence rate (EAIR) of adverse events (AEs) during the open-label treatment phase per 100 subject years. The secondary endpoint was the evaluation of the proportion of patients discontinuing the open-label treatment phase due to AEs and due to non-AE reasons.

Further exploratory endpoints comprised the evaluation of patient satisfaction and quality of Life (QoL) outcome parameters via Treatment Satisfaction Questionnaire for Medication (TSQM VER II) as well as Headache Impact Test (HIT-6) questionnaire, both at week 1, 24, 48, 72, 96, 120, and 128. The domains assessed by TSQM-II include effectiveness, side effects, convenience, and global satisfaction, where every domain is scored 0 − 100. Higher scores indicate higher satisfaction [10]. HIT-6 is a short-form questionnaire based on the internet-HIT question pool [11]. For each HIT-6 item, 6, 8, 10, 11, or 13 points, respectively, are assigned to the response provided and summed to produce a total HIT-6 score that ranges from 36 to 78. HIT-6 scores of ≤ 49 represent little or no impact, of 50 − 55 some impact, of 56 − 59 substantial impact, and of 60 − 78 severe impact due to headache.

### Subgroup analysis of patients with planned drug holiday

Furthermore, the effect of erenumab drug holiday on safety, tolerability and QoL was evaluated. Proportion of patients with drug holiday, time until treatment interruption, duration of treatment interruption as well as proportion of patients returning to treatment scheme after treatment interruption were assessed. Patients with drug holiday documented MMD and monthly headache days (MHD) in a headache diary in the 4 weeks before, during and 12 weeks after the drug holiday.

A significant deterioration in MMD or MHD was defined as an increase in days during the drug holiday of ≥ 30% compared to the number of days in the period of 4 weeks before the drug holiday (the worst value during the drug holiday was used for comparison). A significant improvement after treatment resumption was defined as a decrease in days during the drug holiday of ≥ 30% within 12 weeks after the drug holiday compared to the number of days during the drug holiday (the mean value during the drug holiday and the best value after the drug holiday are used for comparison). For longitudinal comparisons, only patients with at least one monthly interval (with ≥ 14 documented diary days each) during drug holiday initiation and during drug holiday were included in the analysis.

### Data analysis

#### Statistical analysis

Categorical data are presented as frequencies and percentages. In general, missing values were not considered for calculation of percentages (i.e., adjusted percentages are calculated), if not otherwise specified.

For continuous data, mean, standard deviation, median, minimum, and maximum were presented.

Analyses on the primary endpoint were based on the Safety Analysis Set (SAF, all patients who received at least one dose of study treatment in the open-label treatment phase, *N* = 701).

The primary endpoint variable EAIR of AEs during the open-label treatment phase per 100 patient years was calculated by dividing the number of AEs by the time under treatment and standardizing it per 100 patient-years. Exact Pearson-Clopper confidence intervals for single proportions were calculated to evaluate the precision of the estimated parameter. No formal hypotheses testing was conducted. Missing data were not imputed for the primary endpoint.

The secondary endpoints, proportion of patients discontinuing open-label treatment phase due to AE and proportion of patients discontinuing open-label treatment phase due to reasons other than AEs were descriptively analyzed based on the SAF.

Analysis of exploratory efficacy endpoints utilized the Full Analysis Set (FAS, equivalent to SAF, *N* = 701).

Demographics and other baseline characteristics have been collected for all patients, including: age, sex, race, relevant medical history/current medical condition present before signing informed consent for trial HER-MES. These data from HER-MES trial will also be included in the analyses for APOLLON.

## Results

In the preceding HER-MES study, 777 patients were randomized to either receive erenumab (*N* = 389) or topiramate (*N* = 388) [6]. Of these, a total of 701 patients were enrolled in the APOLLON study. Patients had a mean age of 41.8 years and a female representation of 86.7%. Average duration of the disease to date was 22 years and in 66% of the patients, aura was present. On average, participants had 10.4 MMD and the majority of patients (67.1%) had 8 − 14 MMD. Most of the patients had experience with acute migraine medication (81.6%), however, more than half of the participants (57.9%) were naïve to prophylactic treatment. Of those with prior prophylactic medication (*N* = 294), the majority had one treatment failure (31.4% of all participants; two treatment failures: 9.3%; three treatment failures: 1.3%) (Table 1; demographics and other baseline characteristics have been collected during the HER-MES study).

Two thirds of patients started treatment with 140 mg erenumab (66.2%; 70 mg start dose: 33.8%). Over the course of the study, dose increases from 70 to 140 mg were documented for approximately 20% of patients, mainly due to insufficient response as deemed by physician and/or patient, while dose reductions from 140 to 70 mg were documented for about 6% of patients. The average duration of exposure to erenumab was 109.9 ± 35.3 weeks excluding drug holiday with about 70% of patients receiving between 31 and 33 doses of erenumab and a further 10% receiving 26 to 30 doses (**data not shown**).

**Table 1:** Patient baseline characteristics

Patients screened, *N*	702
Patients enrolled, N	701
Age, years ± SD	41.8 ± 12.3
Female, n (%)	608 (86.7)
Disease duration, years ± SD	22 ± 12
Aura present, n (%)	238 (34.0)
Monthly headache days^a^, days ± SD	11.5 ± 4.1
Monthly migraine days^a^, days ± SD	10.4 ± 3.8
Monthly migraine days categories	
*4 − 7 monthly migraine days*,* n (%)*	*165 (23.5)*
*8 − 14 monthly migraine days*,* n (%)*	*470 (67.0)*
*≥ 15 monthly migraine days*,* n (%)*	*65 (9.3)*
Acute headache medication, n (%)	681 (97.1)
*Migraine specific medication*,* n (%)*	*572 (81.6)*
*Non-migraine specific medication*,* n (%)*	*109 (15.5)*
Prior prophylactic treatment failure status^b^	
*Naive*	*406 (57.9)*
*Prior failure*	*294 (41.9)*
*one treatment failed*,* n (%)*	*220 (31.4)*
*two treatment failed*,* n (%)*	*65 (9.3)*
*three treatment failed*,* n (%)*	*9 (1.3)*

^a^Normalized to 28 days. ^b^Prior treatment failure of propranolol/metoprolol, amitriptyline, flunarizine

### Primary safety endpoint – exposure-adjusted incidence rate (EAIR)

The EAIR of AEs during the open-label treatment phase per 100 subject years based on patients with at least one AE (*N* = 601) amounted to 101.71, 95% CI [92.28; 111.14]. This means a patient could expect to have about one adverse event per each year of treatment. The EAIR was higher in females (*n* = 524, EAIR: 104.40, 95% CI [93.93; 114.86]) than in males (*n* = 77, EAIR: 86.55, 95% CI [65.39; 107.71]) and increased with the initial number of MMD as well as in patients with two or three prior prophylactic treatment failures. There were no relevant differences in EAIR between 70 mg and 140 mg erenumab starting doses (Table 2).

### Secondary safety endpoint – treatment discontinuation due to AEs

A total of 155 patients discontinued erenumab treatment during open-label treatment phase. In most cases, treatment discontinuations were not AE-related (*N* = 126, 17.8%, Table 2). The primary reasons were lack of efficacy (*N* = 45) and patient decision (*N* = 44). In 29 subjects (4.1%) who discontinued erenumab treatment due to AEs, the main reasons were diseases of the nervous system (incl. migraine) (*N* = 8), the gastrointestinal tract (incl. constipation) (*N* = 8), the skin and the subcutaneous tissue (*N* = 8), along with other system organ classes.

### General safety − treatment-emergent adverse events (TEAE)

A total of 220 (31.4%) patients experienced AEs that were classified as treatment-related to erenumab (Table 2). Serious treatment-emergent AEs were observed in 3 patients (0.4%), which comprised tachycardia (*N* = 1), spontaneous abortion (*N* = 1) and Raynaud’s phenomenon (*N* = 1). In 62% of the cases, where study treatment was discontinued due to AEs (*N* = 37), AEs were classified as treatment-emergent (*N* = 23). Over the course of the study, a total of 601 (85.7%) patients experienced AEs, with similar rates across erenumab starting doses. Most frequently, patients were affected by COVID-19 (*n* = 243, 34.7%), nasopharyngitis (*n* = 134, 19.1%), constipation (*n* = 103, 14.7%), and fatigue (*n* = 61, 8.7%).

No dose effects were observed with regard to the occurrence of serious adverse events (SAEs). Both groups experienced similar rates of SAEs, affecting a total of 86 patients (12.3%) corresponding to *n* = 28 (11.8%) patients with 70 mg dose and *n* = 58 (12.5%) patients with 140 mg dose. Of all SAEs, only a small proportion were treatment-related (*N* = 3, 0.4%) or led to study discontinuation (*N* = 3, 0.4%). No deaths were reported in the study (Table 2).

**Table 2:** Evaluation of safety (study endpoints and general safety)

	*n*	EAIR [CI]
**EAIR of AEs during the open-label treatment phase per 100 subject years**	601	101.71 [92.28; 111.14]
**EAIR of AEs during open-label treatment phase/100 subject years/subgroup**		
Gender		
*Female*	*524*	*104.40 [93.93; 114.86]*
*Male*	*77*	*86.55 [65.39; 107.71]*
Monthly migraine days (MMD)		
*4 − 7 MMDs*	*143*	*95.92 [78.39; 113.45]*
*8 − 14 MMDs*	*402*	*101.11 [89.59; 112.64]*
*≥ 15 MMDs*	*55*	*124.98 [84.25; 165.71]*
Starting dose		
*70 mg*	*205*	*103.35 [86.34; 120.37]*
*140 mg*	*396*	*100.88 [89.57; 112.19]*
Prior prophylactic treatment failure status		
*Naive*	*339*	*99.32 [86.81; 111.83]*
*Prior prophylactic treatment failure (1 − 3 treatments)*	*262*	*104.98 [90.64; 119.31]*
*1 treatment failed*	*193*	*98.30 [83.25; 113.34]*
*2 treatments failed*	*59*	*125.38 [84.64; 166.12]*
*3 treatments failed*	*9*	*151.49 [14.23; 288.75]*
Patients discontinuing open-label treatment phase*	*n* = 155	*N* = 701
Reason for discontinuation	n (%)	N (%)
*Other (non-AE)*	*126 (81.3%)*	*126 (17.8%)*
*AEs*	*29 (18.7%)*	*29 (4.1%)*
**General safety − TEAEs occurring during study (Safety Set)**	n (%)	
Category^#^	Total (*N* = 701), n (%)	
*Any AE*	*601 (85.7%)*	
*70 mg Starting dose*	*205 (29.2%)*	
*140 mg starting dose*	*396 (56.5%)*	
*AE affecting ≥ 5% of the patients*		
*COVID-19*	*243 (34.7%)*	
*Nasopharyngitis*	*134 (19.1%)*	
*Constipation*	*103 (14.7%)*	
*Fatigue*	*61 (8.7%)*	
*Hypertension*	*47 (6.7%)*	
*Back pain*	*45 (6.4%)*	
*Migraine*	*45 (6.4%)*	
*Headache*	*43 (6.1%)*	
*Immunization reaction*	*43 (6.1%)*	
*Depression*	*40 (5.7%)*	
*Study treatment related AE*	*220 (31.4%)*	
*AE leading to study treatment discontinuation*	*37 (5.3%)*	
*AE leading to dose adjustment (including study treatment discontinuation)*	*73 (10.4%)*	
*Serious AE (SAE)*	*86 (12.3%)*	
*70 mg*	*28 (11.8%)*	
*140 mg*	*58 (12.5%)*	
*Study treatment related SAE*	*3 (0.4%)*	
*SAE leading to study treatment discontinuation*	*3 (0.4%)*	
*Deaths*	*0*	

*Derived from treatment completion page in eCRF (varies from separate AE documentation). ^#^A patients with multiple occurrences of an AE was counted only once in the AE category. Events are shown that were either ongoing beyond the end of the trial CAMG334ADE01 (HER-MES) or started after. AEs include all, non-serious AEs and SAEs. AE: adverse event, EAIR: exposure-adjusted incidence rate, SAE: serious adverse event, TEAE: Treatment-emergent adverse event

### Quality of life (QoL)

Evaluation of QoL was assessed via HIT-6 and TSQM-II scores. The HIT-6 score improved from a baseline mean score (SD) of 57.5 (7.3) points by 5.5 points after 6 months of treatment (mean score [SD]: 51.9 [8.0]) with ongoing stabilization until the end of treatment (mean score [SD]: 52.1 [8.4]). Two months after the last dose of erenumab, mean HIT-6 scores slightly increased to 53.4 [8.8] points (week 128).

**Fig. 2:**
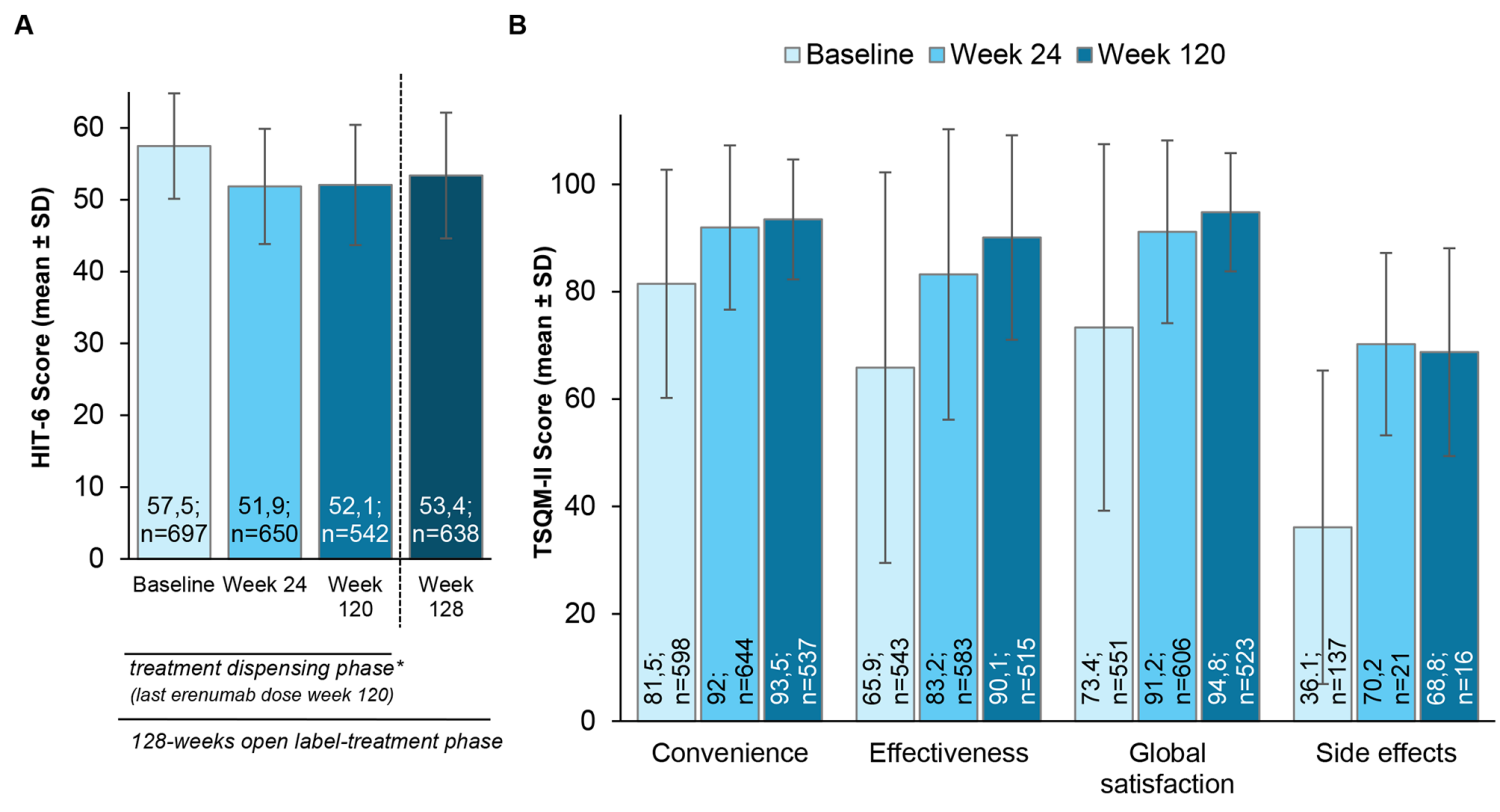
Evaluation of Quality of Life (QoL). Evaluation was assessed via HIT-6 (**A**) and TSQM-II score (**B**) at baseline, after 6 months (M6) and after 30 months (Week 120). *According to the assessment schedule, during open-label treatment phase last erenumab dose was dispensed at Week 120. HIT-6: Headache Impact Test; SD: standard deviation; TSQM: Treatment Satisfaction Questionnaire for Medication

In addition, the TSQM-II scores improved after 6 months of erenumab treatment vs. baseline for a mean of 10.2 points regarding convenience, 15.4 points regarding effectiveness, 16.7 points with respect to global satisfaction, and 32.4 points regarding side effects. TSQM data remained stable or even improved until the end of the study, as in the case of efficacy (increase of 23.5 points vs. baseline) (Fig. 2).

### Drug holiday

Only 108 patients (15.4%) interrupted the study treatment due to drug holiday. Median time until drug holiday was 12.2 months^1^ [min. 3.0; max. 25.0] and median duration of drug holiday was 89 days [min. 27; max. 743 days]. Of these, 70 patients (64.8%) returned to study treatment erenumab after the drug holiday and the majority of patients (88.6%) returned to the dose received prior to the drug holiday. In a few cases (*n* = 3), the dose was decreased after return to study treatment erenumab (Table 3).

**Table 3:** Drug holiday

Variable	*n* [%]
Study population, n	701
Drug holiday planned, n (% of study population)	108 [15.4]
Time until drug holiday in months, mean ± SD [min; median; max]	13.2 ± 5.6 [3; 12; 25]
Returned from holiday, n (% of drug holiday taken)	70 [64.8]
Drug holiday duration in days, mean ± SD [min; median; max]	138 ± 127 [27; 89; 743]
Dose after returning to study treatment^a^	
*Returning to previous dose*,* n (% of returned from drug holiday)*	*62 [88.6]*
*Returning to dose decrease*,* n (% of returned from drug holiday)*	*3 [4.3]*
*Returning to dose increase*,* n (% of returned from drug holiday)*	*0 [0]*

^a^For some patients, information about the dose before or after drug holiday was unavailable

**Fig. 3:**
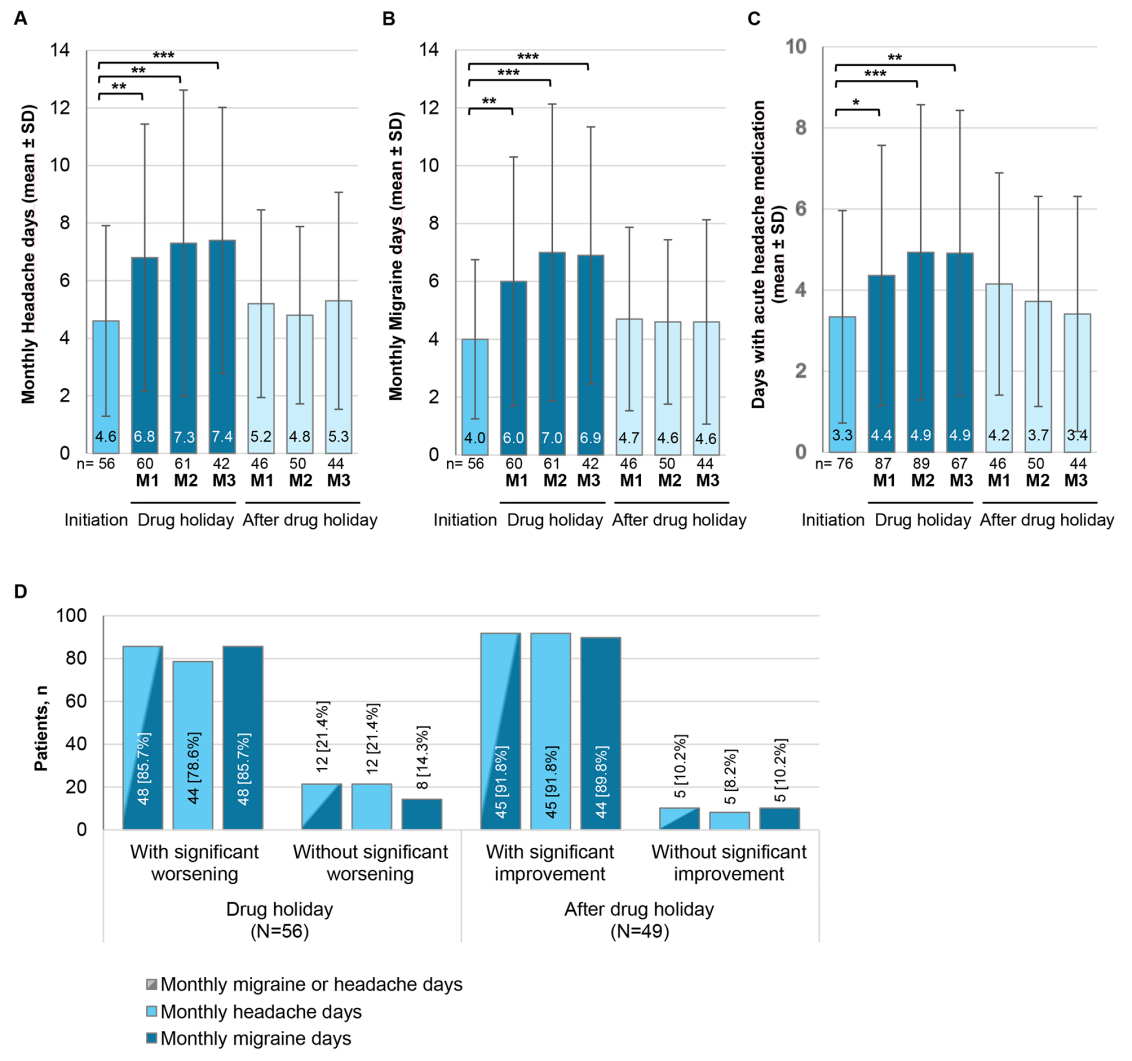
Effects of drug holiday on monthly migraine and headache days and use of acute medication. Monthly headache days (**A**), monthly migraine days (**B**) and days with use of acute medication (**C**) with respect to drug holiday. Number of patients (n), for which headache and migraine diary data was available is indicated in the x-axis. (**D**) Patients with and without significant improvement or deterioration during the drug holiday and after resumption of treatment. Only patients included with at least one monthly interval with ≥ 14 diary days during drug holiday initiation. For the comparison drug holiday initiation to drug holiday (‘drug holiday’), the worst value during drug holiday was used. For the comparison drug holiday to treatment resumption (‘after drug holiday’), the mean value during drug holiday and the best value after drug holiday was used. M1: Month 1; M2: Month 2; M3: Month 3; SD: standard deviation; Wilcoxon test: * *P* ≤ 0.05, ** *P* ≤ 0.01, *** *P* ≤ 0.001, **** *P* ≤ 0.0001 ^a^A significant deterioration is defined as an increase in days during the drug holiday of ≥ 30% compared to the number of days in the period of 4 weeks before the drug holiday (drug holiday initiation phase). The worst value during the drug holiday is used for comparison ^b^Significant improvement after treatment resumption is defined as a decrease in days during the drug holiday of ≥ 30% within 12 weeks after the drug holiday compared to the number of days during the drug holiday. The mean value during the drug holiday and the best value after the drug holiday are used for comparison

Furthermore, the influence of drug holiday on the MHD and MMD as well as on the need for acute medication was assessed. As summarized in Fig. 3, the mean number of MHD and MMD significantly increased during drug holiday, especially in the first and in the second month, reaching a plateau in month 3 (mean difference of 2.8 MHD and 2.7 MMD in month 3 vs. initiation of drug holiday; Wilcoxon test *P* ≤ 0.0001) (Fig. 3, A **− C**). However, after resumption of erenumab treatment, a rapid reduction of MHD and MMD was observed with a return to values similar to those before the drug holiday. Analogously, days with acute headache medication increased by about two days during the drug holiday and upon erenumab re-uptake decreased to levels comparable to pre drug holiday (Fig. 3, A **− C**). Longitudinal analysis of individual patients was performed to assess individual improvement or worsening of disease (change in MMD or MHD of ≥ 30%). Only patients with at least one monthly interval with ≥ 14 diary days each during drug holiday initiation were included. Of the 56 patients with documented headache diary, *N* = 48 (85.7%) showed significant worsening of MHD or MMD during the drug holiday (*N* = 44 [78.6%] had significant worsening of MHD and *N* = 48 [85.7%] of MMD), while *N* = 12 patients (21.4%) had no significant worsening. Upon treatment resumption, *N* = 45 patients (91.8%) with available headache diary (in total *N* = 49) experienced significant improvement of MHD or MMD and only *N* = 5 patients (10.2%) had no significant improvement of MHD or MMD (Fig. 3D).

## Discussion

APOLLON was designed to evaluate the long-term safety and tolerability data on erenumab in patients with EM and CM. Results of the APOLLON study show that the tolerability of erenumab observed in HER-MES over the 24-week period [6] is maintained over the longer period of 128 weeks.

Baseline characteristics of the study population were obtained from the preceding 24-week HER-MES study [6]. At HER-MES baseline, the participants had an average (SD) of 10.4 (3.9) MMDs, and most of the patients had experience with acute migraine medications. In contrast, less than half of the participants had previously used prophylactic treatment (most of these with one treatment failure).

The recommended erenumab dose is 70 mg. However, some patients might benefit from the 140 mg dose [12]. In APOLLON, two thirds of patients started treatment with the higher erenumab dose, which resembled the last dose they have received during preceding HER-MES study, and dose reductions were rare (< 5%). More than half of the patients on the lower starting dose switched to the 140 mg dose during the study, mainly due to insufficient response as deemed by physician and/or patient. Dose changes were permitted within the study protocol and no negative impact on tolerability and safety was observed.

The majority of patients remained adherent, as about 70% of the patients were in the subgroup that received 31 − 33 doses and the dosing regimen allowed a maximum of 33 erenumab doses over the study period of 128 weeks.

The primary endpoint of APOLLON was the EAIR of AEs during the open-label treatment phase per 100 subject years, which was based on 601 patients having at least one AE. The EAIR per 100 subject years amounted to 101.71 (95% CI [92.28; 111.14]) indicating a low frequency of about one AE per 100 patients over one year of treatment. The EAIR was noticeably higher in females than in males. However, this can be explained by females having a much higher migraine disease burden than men [13]. Accordingly, the EAIR increased with the MMD at baseline and with previous treatment failures. There were no relevant differences in EAIR between 70 mg and 140 mg erenumab starting doses. In general, the erenumab EAIR of AEs is rather low. A recent study evaluating long-term efficacy and safety of erenumab in migraine prevention observed an EAIR of 123 in the erenumab open-label extension phase of the study. Although EAIR was higher in the double-blind study phase (placebo vs. erenumab) of the same study, the EAIR was still lower than in the corresponding placebo arm [14]. It should be noted, however, that comparability may be limited as only episodic migraine was analyzed in the aforementioned study by Ashina et al. 2021.

The secondary endpoint of APOLLON was the number of AE-related treatment discontinuations. Only 29 (4.1%) of the 701 patients discontinued erenumab treatment due to AEs and this ongoing low rate of discontinuations confirms the safety profile observed in the HER-MES study [6].

In the APOLLON study, AEs were similar to those commonly reported in other clinical studies and literature (e.g. nasopharingits). However, it should be noted, that the study was conducted during the COVID-19 pandemic, which is reflected by the high number of patients with a related AE (243 patients were affected by Covid-19). This might be a potential factor influencing the study results as well as patient compliance.

Over the course of the study, a total of 85.7% of patients experienced AEs, with similar rates across erenumab starting doses. In general, the rate of erenumab treatment-related TEAE was low and only rarely led to termination of the study (0.4% of the patients).

The overall safety and tolerability profile based on the phase 2 and phase 3 erenumab study program is similar to placebo for both doses [3–5]. APOLLON data add to the favorable long-term safety profile as no new safety signals were observed.

QoL assessment revealed clinically relevant lowering of HIT-6 scores by a mean (SD) of 5.5 (7.8) points after 6 months with ongoing stabilization until the end of the study. These results complement the results already observed in the preceding HER-MES study [6] and point to sustained improvement of QoL under long-term erenumab treatment. This is also reflected by the lasting improvements of the TSQM-II including convenience, effectiveness, global satisfaction, and side effects, which were all improved early (at 6 months) and remained so until the end of the study.

Patients were allowed to take a single drug holiday of up to 24 weeks. This is in line with guideline recommendations, which suggest considering a pause in the treatment with monoclonal antibodies targeting the CGRP pathway after 12 − 18 months of continuous treatment (although treatment should be continued as long as deemed necessary). Restarting of the treatment is suggested if migraine worsens after treatment withdrawal [7]. In APOLLON, only 108 patients (15.4%) paused the study treatment during the 32 months treatment phase for a median of half the per protocol possible time (12.7/24 weeks). As patients’ quality of life parameters improve over the course of the study, it might be conceivable that the patients did not want to risk a migraine relapse.

Two thirds of the patients with interruption of erenumab treatment for the reason of drug holiday returned to the APOLLON study and the vast majority also returned to their prior erenumab dose. Expectedly, the mean number of MHD, MMD, and days with acute headache medication significantly increased during drug holiday, especially in the first two months, reaching a plateau in month 3. However, resumption of erenumab treatment resulted in rapid return to pre-holiday values. Of note, baseline MHDs and MMDs (see Table 1 and HER-MES baseline data [6]) were still higher compared to their increase during the drug holidays.

Longitudinal analyses of individual patients showed that the majority of patients had an increase in MMD/MHD of ≥ 30% compared to 4 weeks before drug holiday and decrease for ≥ 30% within 12 weeks after the drug holiday upon treatment resumption. Also, there was a subgroup of patients (21.4%) that remained stable during the drug holiday. This is in line with data reported by others showing migraine attack rebound early after treatment interruption, but similarly early reduction of MHDs and use of acute medication in response to treatment resumption [15, 16]. Similar results were observed for other CGRP antibodies [17, 18]. Likewise, some patients seem to respond more long-term during treatment pause than others and predictors of drug holiday responses remain to be explored.

Indication for preventive migraine management with the aim to decrease attack frequency, duration, and severity, results from particular suffering, restriction of quality of life and the risk of overuse of medication [16]. Patients benefited long-term from erenumab treatment during the APOLLON study regarding these aspects. A specific timepoint for drug discontinuation attempt and/or duration of interruption of 3 months is not supported by the presented data. Rather a fixed drug interruption of 3 months should be challenged, and individualizing patients’ prophylactic management should be supported. Overall, if it is necessary to resume treatment, most patients show similar response rates compared to initial treatment, which might reduce patients’ fear of taking a drug discontinuation attempt.

## Conclusions

To date, only limited data are available regarding long-term safety and tolerability of erenumab therapy, especially when considering dose adjustments and drug holidays more closely resembling the real-world setting and guideline recommendations. Meanwhile, treatment holidays are recommended by the European Headache Federation guideline upon use of monoclonal antibodies targeting the CGRP pathway [7]. The APOLLON study provides long-term safety and tolerability data confirming the known safety profile of erenumab. In addition, reversibility of migraine deterioration during drug holiday was shown with the majority of patients returning to their treatment with similar response rates to initial treatment.

## Data Availability

The study data for the analysis described in this report may be made available on request by the author investigators or Novartis Pharma GmbH, sponsor of this clinical research.

## Footnotes

1 1 month = 28 days.

## References

[1] Porst M, Wengler A, Leddin J et al (2020) Migraine and tension-type headache in Germany. Prevalence and disease severity from the BURDEN 2020 Burden of Disease Study. J Health Monit 5(Suppl 6):2–24. 10.25646/6990.2 35146296 10.25646/6990.2 PMC8734075

[2] Kawata AK, Shah N, Poon JL et al (2021) Understanding the migraine treatment landscape prior to the introduction of calcitonin gene-related peptide inhibitors: results from the Assessment of TolerabiliTy and Effectiveness in MigrAINe patients using Preventive Treatment (ATTAIN) study. Headache 61(3):438–454. 10.1111/head.14053 33594686 10.1111/head.14053 PMC8048891

[3] Goadsby PJ, Reuter U, Hallstrom Y et al (2017) A controlled trial of Erenumab for episodic migraine. N Engl J Med 377(22):2123–2132. 10.1056/NEJMoa1705848 29171821 10.1056/NEJMoa1705848

[4] Reuter U, Goadsby PJ, Lanteri-Minet M et al (2018) Efficacy and tolerability of erenumab in patients with episodic migraine in whom two-to-four previous preventive treatments were unsuccessful: a randomised, double-blind, placebo-controlled, phase 3b study. Lancet 392(10161):2280–2287. 10.1016/S0140-6736(18)32534-0 30360965 10.1016/S0140-6736(18)32534-0

[5] Dodick DW, Ashina M, Brandes JL et al (2018) ARISE: a phase 3 randomized trial of erenumab for episodic migraine. Cephalalgia 38(6):1026–1037. 10.1177/0333102418759786 29471679 10.1177/0333102418759786

[6] Reuter U, Ehrlich M, Gendolla A et al (2022) Erenumab versus topiramate for the prevention of migraine - a randomised, double-blind, active-controlled phase 4 trial. Cephalalgia 42(2):108–118. 10.1177/03331024211053571 34743579 10.1177/03331024211053571 PMC8793299

[7] Sacco S, Amin FM, Ashina M et al (2022) European Headache Federation guideline on the use of monoclonal antibodies targeting the calcitonin gene related peptide pathway for migraine prevention – 2022 update. J Headache Pain 23(1):67. 10.1186/s10194-022-01431-x 35690723 10.1186/s10194-022-01431-x PMC9188162

[8] Ashina M, Tepper S, Brandes JL et al (2018) Efficacy and safety of erenumab (AMG334) in chronic migraine patients with prior preventive treatment failure: a subgroup analysis of a randomized, double-blind, placebo-controlled study. Cephalalgia 38(10):1611–1621. 10.1177/0333102418788347 29984601 10.1177/0333102418788347

[9] Tepper SJ, Sheikh HU, Dougherty CO et al (2022) Erenumab dosage for migraine prevention: an evidence-based narrative review with recommendations. Headache 62(4):420–435. 10.1111/head.14266 35137404 10.1111/head.14266

[10] Atkinson MJ, Kumar R, Cappelleri JC et al (2005) Hierarchical construct validity of the treatment satisfaction questionnaire for medication (TSQM version II) among outpatient pharmacy consumers. Value Health 8 Suppl 1S9–S24. 10.1111/j.1524-4733.2005.00066.x 10.1111/j.1524-4733.2005.00066.x 16336491

[11] Kosinski M, Bayliss MS, Bjorner JB et al (2003) A six-item short-form survey for measuring headache impact: the HIT-6. Qual Life Res 12(8):963–974. 10.1023/a:1026119331193 14651415 10.1023/a:1026119331193

[12] Fachinformation Aimovig^®^ 70 mg / 140 mg Injektionslösung im Fertigpen Rote Liste (2023) Arzneimittelverzeichnis für Deutschland (einschließlich EU-Zulassungen und bestimmter Medizinprodukte). Rote Liste Service GmbH (Verlag). ISBN 978-3-946057-84-0

[13] Chalmer MA, Kogelman LJA, Callesen I et al (2023) Sex differences in clinical characteristics of migraine and its burden: a population-based study. Eur J Neurol 30(6):1774–1784. 10.1111/ene.15778 36905094 10.1111/ene.15778

[14] Ashina M, Goadsby PJ, Reuter U et al (2021) Long-term efficacy and safety of erenumab in migraine prevention: results from a 5-year, open-label treatment phase of a randomized clinical trial. Eur J Neurol 28(5):1716–1725. 10.1111/ene.14715 33400330 10.1111/ene.14715 PMC8248354

[15] De Matteis E, Affaitati G, Frattale I et al (2021) Early outcomes of migraine after erenumab discontinuation: data from a real-life setting. Neurol Sci 42(8):3297–3303. 10.1007/s10072-020-05022-z 33389227 10.1007/s10072-020-05022-z

[16] Gossrau G, Forderreuther S, Ruscheweyh R et al (2023) [Consensus statement of the migraine and headache societies (DMKG, OKSG, and SKG) on the duration of pharmacological migraine prophylaxis]. Nervenarzt 94(4):306–317. 10.1007/s00115-022-01403-1 36287216 10.1007/s00115-022-01403-1 PMC9607745

[17] Raffaelli B, Terhart M, Mecklenburg J et al (2022) Resumption of migraine preventive treatment with CGRP(-receptor) antibodies after a 3-month drug holiday: a real-world experience. J Headache Pain 23(1):40. 10.1186/s10194-022-01417-9 35350990 10.1186/s10194-022-01417-9 PMC8966337

[18] Raffaelli B, Terhart M, Overeem LH et al (2022) Migraine evolution after the cessation of CGRP(-receptor) antibody prophylaxis: a prospective, longitudinal cohort study. Cephalalgia 42(4–5):326–334. 10.1177/03331024211046617 34579559 10.1177/03331024211046617 PMC8988461

